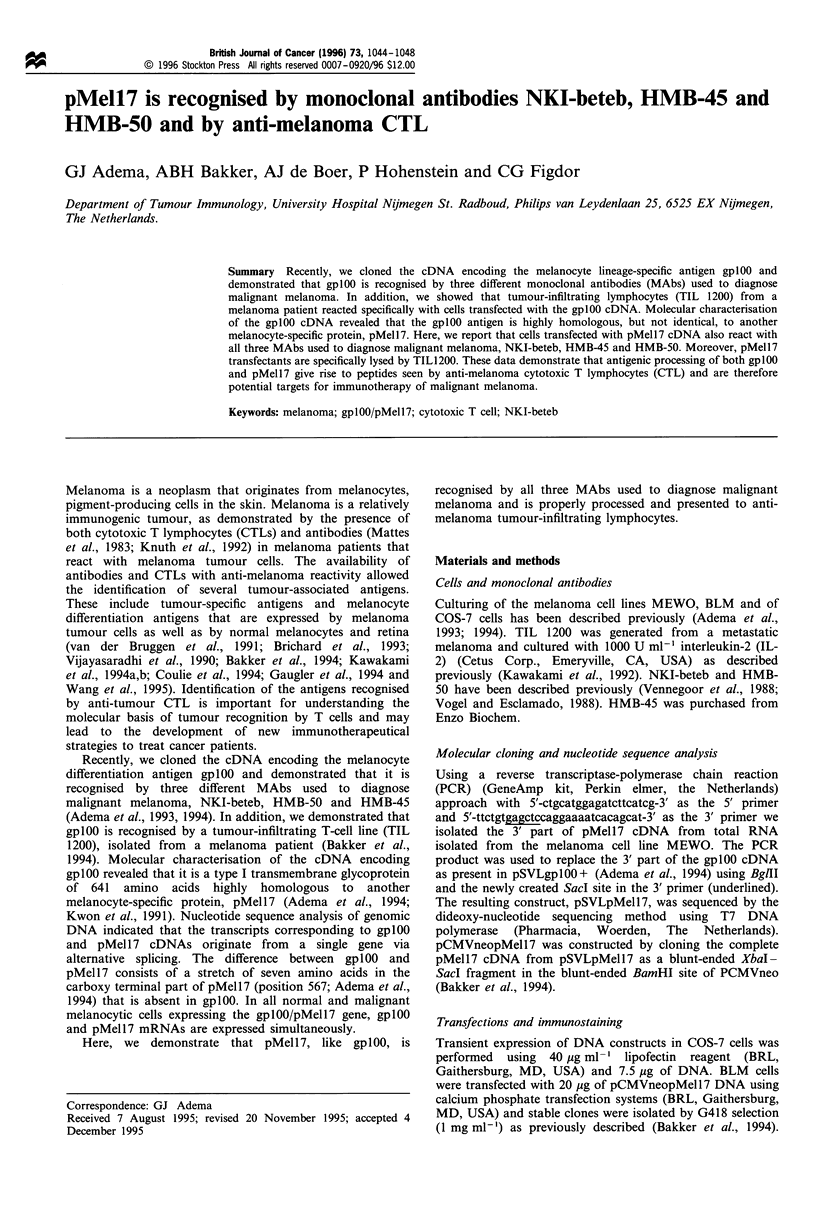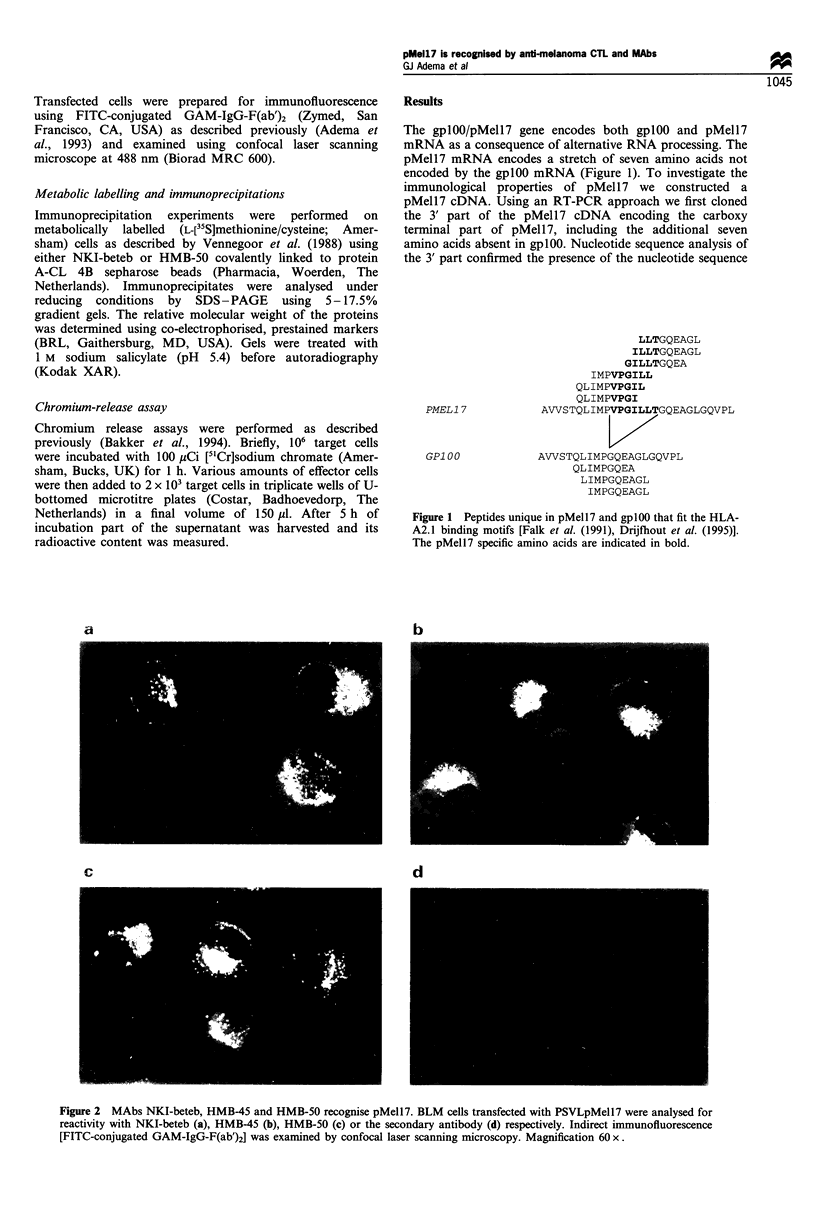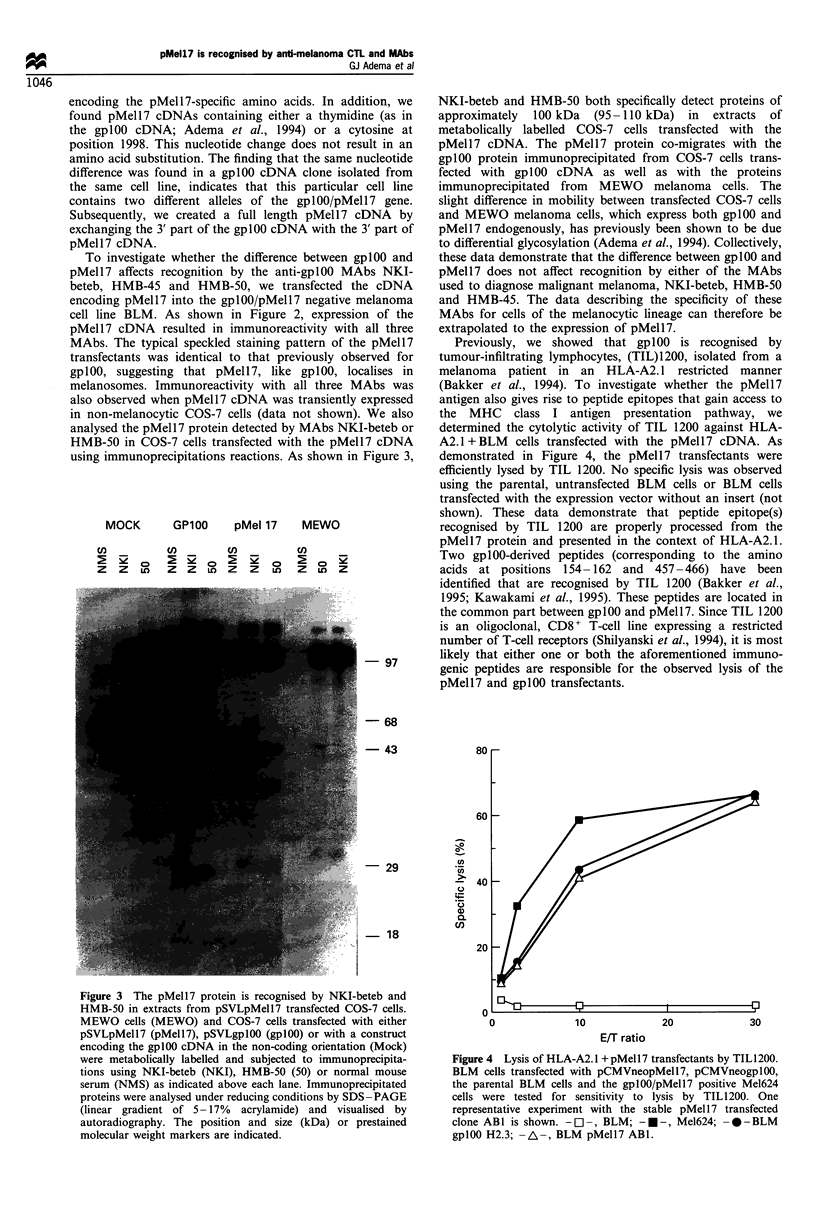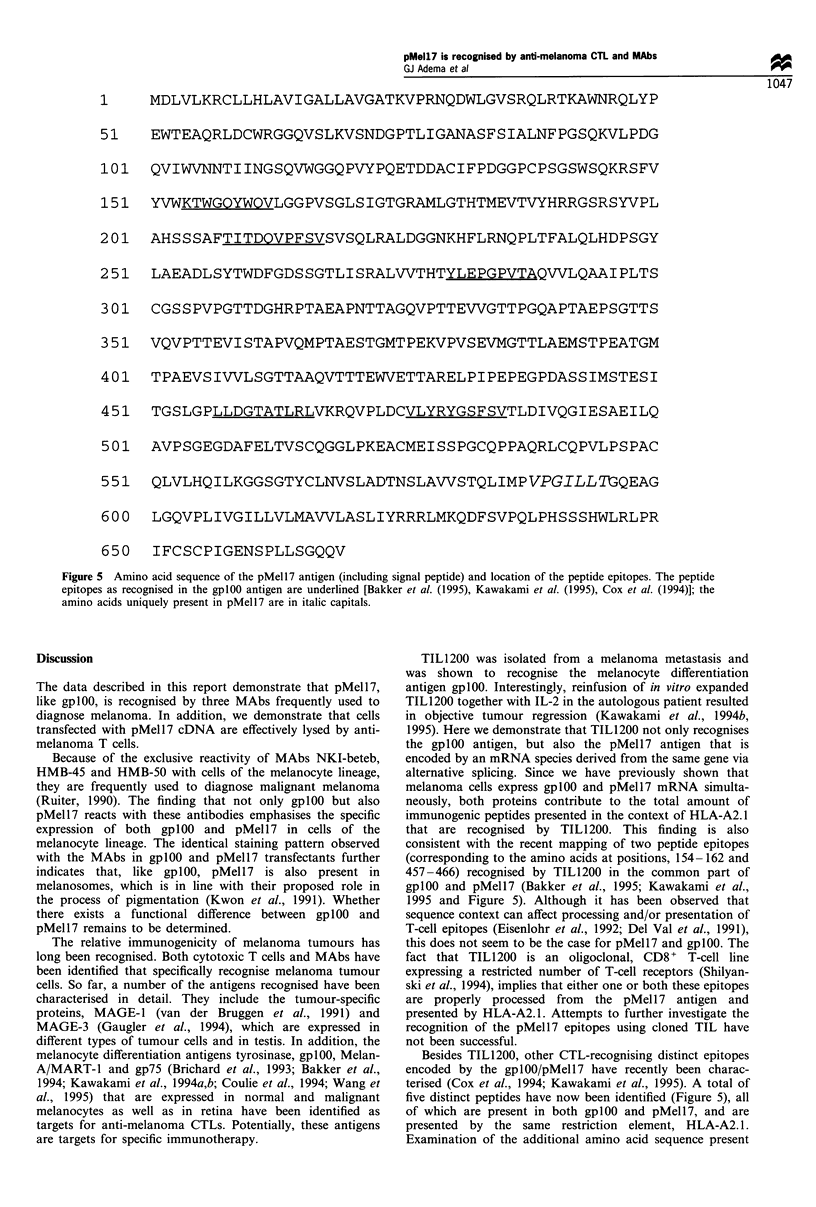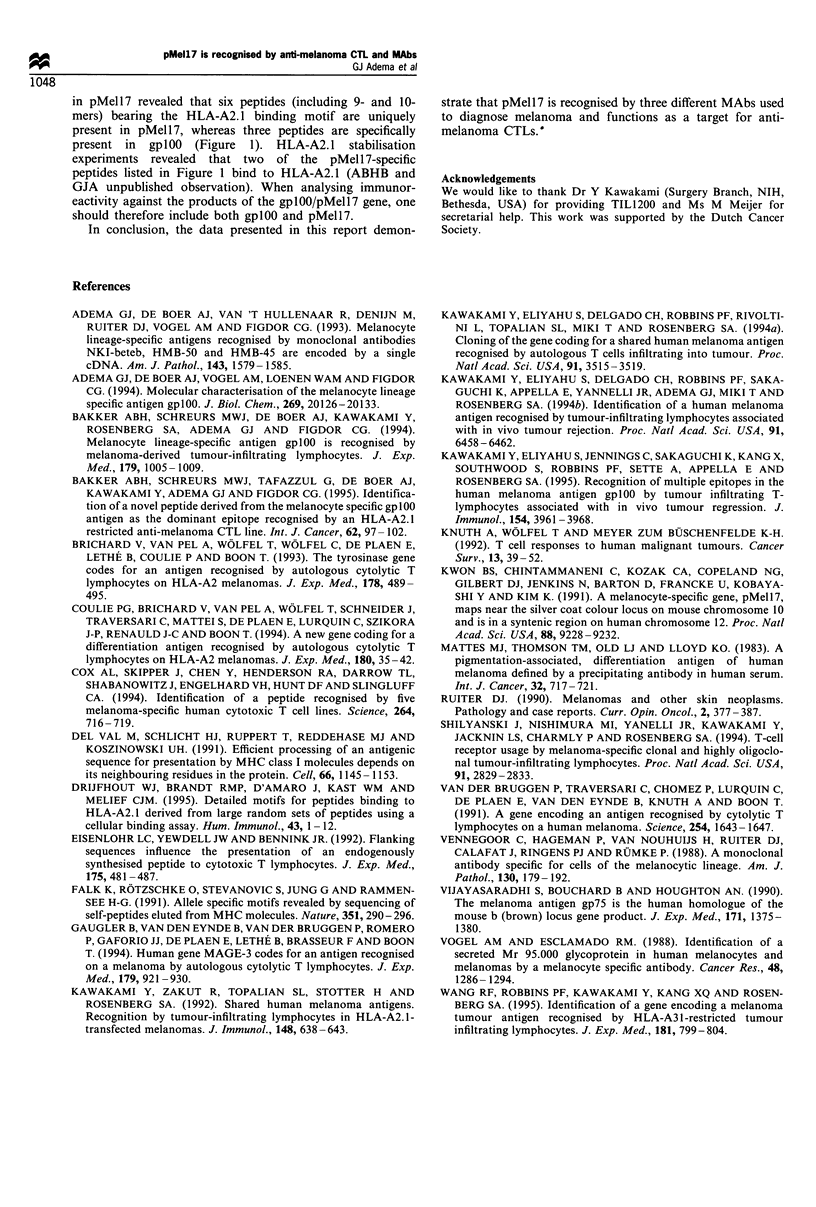# pMel17 is recognised by monoclonal antibodies NKI-beteb, HMB-45 and HMB-50 and by anti-melanoma CTL.

**DOI:** 10.1038/bjc.1996.202

**Published:** 1996-05

**Authors:** G. J. Adema, A. B. Bakker, A. J. de Boer, P. Hohenstein, C. G. Figdor

**Affiliations:** Department of Tumour Immunology, University Hospital Nijmegen St. Radboud, The Netherlands.

## Abstract

**Images:**


British Journal of Cancer (1996) 73, 1044–1048
© 1996 Stockton Press All rights reserved 0007–0920/96 $12.00

### pMel17 is recognised by monoclonal antibodies NKI-beteb, HMB-45 and HMB-50 and by anti-melanoma CTL

GJ Adema, ABH Bakker, AJ de Boer, P Hohenstein and CG Figdor

*Department of Tumour Immunology, University Hospital Nijmegen St. Radboud, Philips van Leydenlaan 25, 6525 EX Nijmegen, The Netherlands.*

**Summary** Recently, we cloned the cDNA encoding the melanocyte lineage-specific antigen gp100 and demonstrated that gp100 is recognised by three different monoclonal antibodies (MAbs) used to diagnose malignant melanoma. In addition, we showed that tumour-infiltrating lymphocytes (TIL 1200) from a melanoma patient reacted specifically with cells transfected with the gp100 cDNA. Molecular characterisation of the gp100 cDNA revealed that the gp100 antigen is highly homologous, but not identical, to another melanocyte-specific protein, pMel17. Here, we report that cells transfected with pMel17 cDNA also react with all three MAbs used to diagnose malignant melanoma, NKI-beteb, HMB-45 and HMB-50. Moreover, pMel17 transfectants are specifically lysed by TIL1200. These data demonstrate that antigenic processing of both gp100 and pMel17 give rise to peptides seen by anti-melanoma cytotoxic T lymphocytes (CTL) and are therefore potential targets for immunotherapy of malignant melanoma.

**Keywords:** melanoma; gp100/pMel17; cytotoxic T cell; NKI-beteb

Melanoma is a neoplasm that originates from melanocytes, pigment-producing cells in the skin. Melanoma is a relatively immunogenic tumour, as demonstrated by the presence of both cytotoxic T lymphocytes (CTLs) and antibodies (Mattes *et al.*, 1983; Knuth *et al.*, 1992) in melanoma patients that react with melanoma tumour cells. The availability of antibodies and CTLs with anti-melanoma reactivity allowed the identification of several tumour-associated antigens. These include tumour-specific antigens and melanocyte differentiation antigens that are expressed by melanoma tumour cells as well as by normal melanocytes and retina (van der Bruggen *et al.*, 1991; Brichard *et al.*, 1993; Vijayasaradhi *et al.*, 1990; Bakker *et al.*, 1994; Kawakami *et al.*, 1994a,b; Coulie *et al.*, 1994; Gaugler *et al.*, 1994 and Wang *et al.*, 1995). Identification of the antigens recognised by anti-tumour CTL is important for understanding the molecular basis of tumour recognition by T cells and may lead to the development of new immunotherapeutical strategies to treat cancer patients.

Recently, we cloned the cDNA encoding the melanocyte differentiation antigen gp100 and demonstrated that it is recognised by three different MAbs used to diagnose malignant melanoma, NKI-beteb, HMB-50 and HMB-45 (Adema *et al.*, 1993, 1994). In addition, we demonstrated that gp100 is recognised by a tumour-infiltrating T-cell line (TIL 1200), isolated from a melanoma patient (Bakker *et al.*, 1994). Molecular characterisation of the cDNA encoding gp100 revealed that it is a type I transmembrane glycoprotein of 641 amino acids highly homologous to another melanocyte-specific protein, pMel17 (Adema *et al.*, 1994; Kwon *et al.*, 1991). Nucleotide sequence analysis of genomic DNA indicated that the transcripts corresponding to gp100 and pMel17 cDNAs originate from a single gene via alternative splicing. The difference between gp100 and pMel17 consists of a stretch of seven amino acids in the carboxy terminal part of pMel17 (position 567; Adema *et al.*, 1994) that is absent in gp100. In all normal and malignant melanocytic cells expressing the gp100/pMel17 gene, gp100 and pMel17 mRNAs are expressed simultaneously.

Here, we demonstrate that pMel17, like gp100, is recognised by all three MAbs used to diagnose malignant melanoma and is properly processed and presented to anti-melanoma tumour-infiltrating lymphocytes.

Correspondence: GJ Adema
Received 7 August 1995; revised 20 November 1995; accepted 4 December 1995

### Materials and methods

### *Cells and monoclonal antibodies*

Culturing of the melanoma cell lines MEWO, BLM and of COS-7 cells has been described previously (Adema *et al.*, 1993; 1994). TIL 1200 was generated from a metastatic melanoma and cultured with 1000 U $ml^{-1}$ interleukin-2 (IL-2) (Cetus Corp., Emeryville, CA, USA) as described previously (Kawakami *et al.*, 1992). NKI-beteb and HMB-50 have been described previously (Vennegoor *et al.*, 1988; Vogel and Esclamado, 1988). HMB-45 was purchased from Enzo Biochem.

### *Molecular cloning and nucleotide sequence analysis*

Using a reverse transcriptase-polymerase chain reaction (PCR) (GeneAmp kit, Perkin elmer, the Netherlands) approach with 5′-ctgcatggagatcttcatcg-3′ as the 5′ primer and 5′-ttctgtgagctccaggaaaatcacagcat-3′ as the 3′ primer we isolated the 3′ part of pMel17 cDNA from total RNA isolated from the melanoma cell line MEWO. The PCR product was used to replace the 3′ part of the gp100 cDNA as present in pSVLgp100+ (Adema *et al.*, 1994) using *Bgl*II and the newly created *Sac*I site in the 3′ primer (underlined). The resulting construct, pSVLpMel17, was sequenced by the dideoxy-nucleotide sequencing method using T7 DNA polymerase (Pharmacia, Woerden, The Netherlands). pCMVneopMel17 was constructed by cloning the complete pMel17 cDNA from pSVLpMel17 as a blunt-ended *Xba*I–*Sac*I fragment in the blunt-ended *Bam*HI site of PCMVneo (Bakker *et al.*, 1994).

### *Transfections and immunostaining*

Transient expression of DNA constructs in COS-7 cells was performed using 40 $\mu g\ ml^{-1}$ lipofectin reagent (BRL, Gaithersburg, MD, USA) and 7.5 $\mu g$ of DNA. BLM cells were transfected with 20 $\mu g$ of pCMVneopMel17 DNA using calcium phosphate transfection systems (BRL, Gaithersburg, MD, USA) and stable clones were isolated by G418 selection (1 mg $ml^{-1}$) as previously described (Bakker *et al.*, 1994).

Transfected cells were prepared for immunofluorescence using FITC-conjugated GAM-IgG-F(ab′)$_2$ (Zymed, San Francisco, CA, USA) as described previously (Adema *et al*., 1993) and examined using confocal laser scanning microscope at 488 nm (Biorad MRC 600).

### *Metabolic labelling and immunoprecipitations*

Immunoprecipitation experiments were performed on metabolically labelled (L-[$^{35}$S]methionine/cysteine; Amersham) cells as described by Vennegoor *et al.* (1988) using either NKI-beteb or HMB-50 covalently linked to protein A-CL 4B sepharose beads (Pharmacia, Woerden, The Netherlands). Immunoprecipitates were analysed under reducing conditions by SDS–PAGE using 5–17.5% gradient gels. The relative molecular weight of the proteins was determined using co-electrophorised, prestained markers (BRL, Gaithersburg, MD, USA). Gels were treated with 1 M sodium salicylate (pH 5.4) before autoradiography (Kodak XAR).

### *Chromium-release assay*

Chromium release assays were performed as described previously (Bakker *et al.*, 1994). Briefly, $10^6$ target cells were incubated with 100 $\mu$Ci [$^{51}$Cr]sodium chromate (Amersham, Bucks, UK) for 1 h. Various amounts of effector cells were then added to $2 \times 10^3$ target cells in triplicate wells of U-bottomed microtitre plates (Costar, Badhoevedorp, The Netherlands) in a final volume of 150 $\mu$l. After 5 h of incubation part of the supernatant was harvested and its radioactive content was measured.

### Results

The gp100/pMel17 gene encodes both gp100 and pMel17 mRNA as a consequence of alternative RNA processing. The pMel17 mRNA encodes a stretch of seven amino acids not encoded by the gp100 mRNA (Figure 1). To investigate the immunological properties of pMel17 we constructed a pMel17 cDNA. Using an RT-PCR approach we first cloned the 3′ part of the pMel17 cDNA encoding the carboxy terminal part of pMel17, including the additional seven amino acids absent in gp100. Nucleotide sequence analysis of the 3′ part confirmed the presence of the nucleotide sequence

**LLT**GQEAGL
**ILLT**GQEAGL
**GILLT**GQEA
IMP**VPGILL**
QLIMP**VPGIL**
QLIMP**VPGI**
*PMEL17* AVVSTQLIMP**VPGILLT**GQEAGLGQVPL

*GP100* AVVSTQLIMPGQEAGLGQVPL
QLIMPGQEA
LIMPGQEAGL
IMPGQEAGL

**Figure 1** Peptides unique in pMel17 and gp100 that fit the HLA-A2.1 binding motifs [Falk *et al.* (1991), Drijfhout *et al.* (1995)]. The pMel17 specific amino acids are indicated in bold.

**Figure 2** MAbs NKI-beteb, HMB-45 and HMB-50 recognise pMel17. BLM cells transfected with PSVLpMel17 were analysed for reactivity with NKI-beteb (**a**), HMB-45 (**b**), HMB-50 (**c**) or the secondary antibody (**d**) respectively. Indirect immunofluorescence [FITC-conjugated GAM-IgG-F(ab′)$_2$] was examined by confocal laser scanning microscopy. Magnification 60×.

encoding the pMel17-specific amino acids. In addition, we found pMel17 cDNAs containing either a thymidine (as in the gp100 cDNA; Adema *et al.*, 1994) or a cytosine at position 1998. This nucleotide change does not result in an amino acid substitution. The finding that the same nucleotide difference was found in a gp100 cDNA clone isolated from the same cell line, indicates that this particular cell line contains two different alleles of the gp100/pMel17 gene. Subsequently, we created a full length pMel17 cDNA by exchanging the 3′ part of the gp100 cDNA with the 3′ part of pMel17 cDNA.

To investigate whether the difference between gp100 and pMel17 affects recognition by the anti-gp100 MAbs NKI-beteb, HMB-45 and HMB-50, we transfected the cDNA encoding pMel17 into the gp100/pMel17 negative melanoma cell line BLM. As shown in Figure 2, expression of the pMel17 cDNA resulted in immunoreactivity with all three MAbs. The typical speckled staining pattern of the pMel17 transfectants was identical to that previously observed for gp100, suggesting that pMel17, like gp100, localises in melanosomes. Immunoreactivity with all three MAbs was also observed when pMel17 cDNA was transiently expressed in non-melanocytic COS-7 cells (data not shown). We also analysed the pMel17 protein detected by MAbs NKI-beteb or HMB-50 in COS-7 cells transfected with the pMel17 cDNA using immunoprecipitations reactions. As shown in Figure 3, NKI-beteb and HMB-50 both specifically detect proteins of approximately 100 kDa (95–110 kDa) in extracts of metabolically labelled COS-7 cells transfected with the pMel17 cDNA. The pMel17 protein co-migrates with the gp100 protein immunoprecipitated from COS-7 cells transfected with gp100 cDNA as well as with the proteins immunoprecipitated from MEWO melanoma cells. The slight difference in mobility between transfected COS-7 cells and MEWO melanoma cells, which express both gp100 and pMel17 endogenously, has previously been shown to be due to differential glycosylation (Adema *et al.*, 1994). Collectively, these data demonstrate that the difference between gp100 and pMel17 does not affect recognition by either of the MAbs used to diagnose malignant melanoma, NKI-beteb, HMB-50 and HMB-45. The data describing the specificity of these MAbs for cells of the melanocytic lineage can therefore be extrapolated to the expression of pMel17.

Previously, we showed that gp100 is recognised by tumour-infiltrating lymphocytes, (TIL)1200, isolated from a melanoma patient in an HLA-A2.1 restricted manner (Bakker *et al.*, 1994). To investigate whether the pMel17 antigen also gives rise to peptide epitopes that gain access to the MHC class I antigen presentation pathway, we determined the cytolytic activity of TIL 1200 against HLA-A2.1+BLM cells transfected with the pMel17 cDNA. As demonstrated in Figure 4, the pMel17 transfectants were efficiently lysed by TIL 1200. No specific lysis was observed using the parental, untransfected BLM cells or BLM cells transfected with the expression vector without an insert (not shown). These data demonstrate that peptide epitope(s) recognised by TIL 1200 are properly processed from the pMel17 protein and presented in the context of HLA-A2.1. Two gp100-derived peptides (corresponding to the amino acids at positions 154–162 and 457–466) have been identified that are recognised by TIL 1200 (Bakker *et al.*, 1995; Kawakami *et al.*, 1995). These peptides are located in the common part between gp100 and pMel17. Since TIL 1200 is an oligoclonal, $CD8^+$ T-cell line expressing a restricted number of T-cell receptors (Shilyanski *et al.*, 1994), it is most likely that either one or both the aforementioned immunogenic peptides are responsible for the observed lysis of the pMel17 and gp100 transfectants.

**Figure 3** The pMel17 protein is recognised by NKI-beteb and HMB-50 in extracts from pSVLpMel17 transfected COS-7 cells. MEWO cells (MEWO) and COS-7 cells transfected with either pSVLpMel17 (pMel17), pSVLgp100 (gp100) or with a construct encoding the gp100 cDNA in the non-coding orientation (Mock) were metabolically labelled and subjected to immunoprecipitations using NKI-beteb (NKI), HMB-50 (50) or normal mouse serum (NMS) as indicated above each lane. Immunoprecipitated proteins were analysed under reducing conditions by SDS–PAGE (linear gradient of 5–17% acrylamide) and visualised by autoradiography. The position and size (kDa) or prestained molecular weight markers are indicated.

**Figure 4** Lysis of HLA-A2.1+pMel17 transfectants by TIL1200. BLM cells transfected with pCMVneopMel17, pCMVneogp100, the parental BLM cells and the gp100/pMel17 positive Mel624 cells were tested for sensitivity to lysis by TIL1200. One representative experiment with the stable pMel17 transfected clone AB1 is shown. –□–, BLM; –■–, Mel624; –●–BLM gp100 H2.3; –△–, BLM pMel17 AB1.

1 MDLVLKRCLLHLAVIGALLAVGATKVPRNQDWLGVSRQLRTKAWNRQLYP

51 EWTEAQRLDCWRGGQVSLKVSNDGPTLIGANASFSIALNFPGSQKVLPDG

101 QVIWVNNTIINGSQVWGGQPVYPQETDDACIFPDGGPCPSGSWSQKRSFV

151 YVWKTWGQYWQVLGGPVSGLSIGTGRAMLGTHTMEVTVYHRRGSRSYVPL

201 AHSSSAFTITDQVPFSVSVSQLRALDGGNKHFLRNQPLTFALQLHDPSGY

251 LAEADLSYTWDFGDSSGTLISRALVVTHTYLEPGPVTAQVVLQAAIPLTS

301 CGSSPVPGTTDGHRPTAEAPNTTAGQVPTTEVVGTTPGQAPTAEPSGTTS

351 VQVPTTEVISTAPVQMPTAESTGMTPEKVPVSEVMGTTLAEMSTPEATGM

401 TPAEVSIVVLSGTTAAQVTTTEWVETTARELPIPEPEGPDASSIMSTESI

451 TGSLGPLLDGTATLRLVKRQVPLDCVLYRYGSFSVTLDIVQGIESAEILQ

501 AVPSGEGDAFELTVSCQGGLPKEACMEISSPGCQPPAQRLCQPVLPSPAC

551 QLVLHQILKGGSGTYCLNVSLADTNSLAVVSTQLIMP*VPGILLT*GQEAG

600 LGQVPLIVGILLVLMAVVLASLIYRRRLMKQDFSVPQLPHSSSHWLRLPR

650 IFCSCPIGENSPLLSGQQV

**Figure 5** Amino acid sequence of the pMel17 antigen (including signal peptide) and location of the peptide epitopes. The peptide epitopes as recognised in the gp100 antigen are underlined [Bakker *et al.* (1995), Kawakami *et al.* (1995), Cox *et al.* (1994)]; the amino acids uniquely present in pMel17 are in italic capitals.

### Discussion

The data described in this report demonstrate that pMel17, like gp100, is recognised by three MAbs frequently used to diagnose melanoma. In addition, we demonstrate that cells transfected with pMel17 cDNA are effectively lysed by anti-melanoma T cells.

Because of the exclusive reactivity of MAbs NKI-beteb, HMB-45 and HMB-50 with cells of the melanocyte lineage, they are frequently used to diagnose malignant melanoma (Ruiter, 1990). The finding that not only gp100 but also pMel17 reacts with these antibodies emphasises the specific expression of both gp100 and pMel17 in cells of the melanocyte lineage. The identical staining pattern observed with the MAbs in gp100 and pMel17 transfectants further indicates that, like gp100, pMel17 is also present in melanosomes, which is in line with their proposed role in the process of pigmentation (Kwon *et al.*, 1991). Whether there exists a functional difference between gp100 and pMel17 remains to be determined.

The relative immunogenicity of melanoma tumours has long been recognised. Both cytotoxic T cells and MAbs have been identified that specifically recognise melanoma tumour cells. So far, a number of the antigens recognised have been characterised in detail. They include the tumour-specific proteins, MAGE-1 (van der Bruggen *et al.*, 1991) and MAGE-3 (Gaugler *et al.*, 1994), which are expressed in different types of tumour cells and in testis. In addition, the melanocyte differentiation antigens tyrosinase, gp100, Melan-A/MART-1 and gp75 (Brichard *et al.*, 1993; Bakker *et al.*, 1994; Kawakami *et al.*, 1994*a,b*; Coulie *et al.*, 1994; Wang *et al.*, 1995) that are expressed in normal and malignant melanocytes as well as in retina have been identified as targets for anti-melanoma CTLs. Potentially, these antigens are targets for specific immunotherapy.

TIL1200 was isolated from a melanoma metastasis and was shown to recognise the melanocyte differentiation antigen gp100. Interestingly, reinfusion of *in vitro* expanded TIL1200 together with IL-2 in the autologous patient resulted in objective tumour regression (Kawakami *et al.*, 1994*b*, 1995). Here we demonstrate that TIL1200 not only recognises the gp100 antigen, but also the pMel17 antigen that is encoded by an mRNA species derived from the same gene via alternative splicing. Since we have previously shown that melanoma cells express gp100 and pMel17 mRNA simultaneously, both proteins contribute to the total amount of immunogenic peptides presented in the context of HLA-A2.1 that are recognised by TIL1200. This finding is also consistent with the recent mapping of two peptide epitopes (corresponding to the amino acids at positions, 154–162 and 457–466) recognised by TIL1200 in the common part of gp100 and pMel17 (Bakker *et al.*, 1995; Kawakami *et al.*, 1995 and Figure 5). Although it has been observed that sequence context can affect processing and/or presentation of T-cell epitopes (Eisenlohr *et al.*, 1992; Del Val *et al.*, 1991), this does not seem to be the case for pMel17 and gp100. The fact that TIL1200 is an oligoclonal, $CD8^+$ T-cell line expressing a restricted number of T-cell receptors (Shilyanski *et al.*, 1994), implies that either one or both these epitopes are properly processed from the pMel17 antigen and presented by HLA-A2.1. Attempts to further investigate the recognition of the pMel17 epitopes using cloned TIL have not been successful.

Besides TIL1200, other CTL-recognising distinct epitopes encoded by the gp100/pMel17 have recently been characterised (Cox *et al.*, 1994; Kawakami *et al.*, 1995). A total of five distinct peptides have now been identified (Figure 5), all of which are present in both gp100 and pMel17, and are presented by the same restriction element, HLA-A2.1. Examination of the additional amino acid sequence present in pMel17 revealed that six peptides (including 9- and 10-mers) bearing the HLA-A2.1 binding motif are uniquely present in pMel17, whereas three peptides are specifically present in gp100 (Figure 1). HLA-A2.1 stabilisation experiments revealed that two of the pMel17-specific peptides listed in Figure 1 bind to HLA-A2.1 (ABHB and GJA unpublished observation). When analysing immunoreactivity against the products of the gp100/pMel17 gene, one should therefore include both gp100 and pMel17.

In conclusion, the data presented in this report demonstrate that pMel17 is recognised by three different MAbs used to diagnose melanoma and functions as a target for anti-melanoma CTLs.*

**Acknowledgements**
We would like to thank Dr Y Kawakami (Surgery Branch, NIH, Bethesda, USA) for providing TIL1200 and Ms M Meijer for secretarial help. This work was supported by the Dutch Cancer Society.

### References

ADEMA GJ, DE BOER AJ, VAN 'T HULLENAAR R, DENIJN M, RUITER DJ, VOGEL AM AND FIGDOR CG. (1993). Melanocyte lineage-specific antigens recognised by monoclonal antibodies NKI-beteb, HMB-50 and HMB-45 are encoded by a single cDNA. *Am. J. Pathol.*, **143**, 1579–1585.

ADEMA GJ, DE BOER AJ, VOGEL AM, LOENEN WAM AND FIGDOR CG. (1994). Molecular characterisation of the melanocyte lineage specific antigen gp100. *J. Biol. Chem.*, **269**, 20126–20133.

BAKKER ABH, SCHREURS MWJ, DE BOER AJ, KAWAKAMI Y, ROSENBERG SA, ADEMA GJ AND FIGDOR CG. (1994). Melanocyte lineage-specific antigen gp100 is recognised by melanoma-derived tumour-infiltrating lymphocytes. *J. Exp. Med.*, **179**, 1005–1009.

BAKKER ABH, SCHREURS MWJ, TAFAZZUL G, DE BOER AJ, KAWAKAMI Y, ADEMA GJ AND FIGDOR CG. (1995). Identification of a novel peptide derived from the melanocyte specific gp100 antigen as the dominant epitope recognised by an HLA-A2.1 restricted anti-melanoma CTL line. *Int. J. Cancer*, **62**, 97–102.

BRICHARD V, VAN PEL A, WÖLFEL T, WÖLFEL C, DE PLAEN E, LETHÉ B, COULIE P AND BOON T. (1993). The tyrosinase gene codes for an antigen recognised by autologous cytolytic T lymphocytes on HLA-A2 melanomas. *J. Exp. Med.*, **178**, 489–495.

COULIE PG, BRICHARD V, VAN PEL A, WÖLFEL T, SCHNEIDER J, TRAVERSARI C, MATTEI S, DE PLAEN E, LURQUIN C, SZIKORA J-P, RENAULD J-C AND BOON T. (1994). A new gene coding for a differentiation antigen recognised by autologous cytolytic T lymphocytes on HLA-A2 melanomas. *J. Exp. Med.*, **180**, 35–42.

COX AL, SKIPPER J, CHEN Y, HENDERSON RA, DARROW TL, SHABANOWITZ J, ENGELHARD VH, HUNT DF AND SLINGLUFF CA. (1994). Identification of a peptide recognised by five melanoma-specific human cytotoxic T cell lines. *Science*, **264**, 716–719.

DEL VAL M, SCHLICHT HJ, RUPPERT T, REDDEHASE MJ AND KOSZINOWSKI UH. (1991). Efficient processing of an antigenic sequence for presentation by MHC class I molecules depends on its neighbouring residues in the protein. *Cell*, **66**, 1145–1153.

DRIJFHOUT WJ, BRANDT RMP, D'AMARO J, KAST WM AND MELIEF CJM. (1995). Detailed motifs for peptides binding to HLA-A2.1 derived from large random sets of peptides using a cellular binding assay. *Hum. Immunol.*, **43**, 1–12.

EISENLOHR LC, YEWDELL JW AND BENNINK JR. (1992). Flanking sequences influence the presentation of an endogenously synthesised peptide to cytotoxic T lymphocytes. *J. Exp. Med.*, **175**, 481–487.

FALK K, RÖTZSCHKE O, STEVANOVIC S, JUNG G AND RAMMENSEE H-G. (1991). Allele specific motifs revealed by sequencing of self-peptides eluted from MHC molecules. *Nature*, **351**, 290–296.

GAUGLER B, VAN DEN EYNDE B, VAN DER BRUGGEN P, ROMERO P, GAFORIO JJ, DE PLAEN E, LETHÉ B, BRASSEUR F AND BOON T. (1994). Human gene MAGE-3 codes for an antigen recognised on a melanoma by autologous cytolytic T lymphocytes. *J. Exp. Med.*, **179**, 921–930.

KAWAKAMI Y, ZAKUT R, TOPALIAN SL, STOTTER H AND ROSENBERG SA. (1992). Shared human melanoma antigens. Recognition by tumour-infiltrating lymphocytes in HLA-A2.1-transfected melanomas. *J. Immunol.*, **148**, 638–643.

KAWAKAMI Y, ELIYAHU S, DELGADO CH, ROBBINS PF, RIVOLTINI L, TOPALIAN SL, MIKI T AND ROSENBERG SA. (1994*a*). Cloning of the gene coding for a shared human melanoma antigen recognised by autologous T cells infiltrating into tumour. *Proc. Natl Acad. Sci. USA*, **91**, 3515–3519.

KAWAKAMI Y, ELIYAHU S, DELGADO CH, ROBBINS PF, SAKAGUCHI K, APPELLA E, YANNELLI JR, ADEMA GJ, MIKI T AND ROSENBERG SA. (1994*b*). Identification of a human melanoma antigen recognised by tumour-infiltrating lymphocytes associated with in vivo tumour rejection. *Proc. Natl Acad. Sci. USA*, **91**, 6458–6462.

KAWAKAMI Y, ELIYAHU S, JENNINGS C, SAKAGUCHI K, KANG X, SOUTHWOOD S, ROBBINS PF, SETTE A, APPELLA E AND ROSENBERG SA. (1995). Recognition of multiple epitopes in the human melanoma antigen gp100 by tumour infiltrating T-lymphocytes associated with in vivo tumour regression. *J. Immunol.*, **154**, 3961–3968.

KNUTH A, WÖLFEL T AND MEYER ZUM BÜSCHENFELDE K-H. (1992). T cell responses to human malignant tumours. *Cancer Surv.*, **13**, 39–52.

KWON BS, CHINTAMMANENI C, KOZAK CA, COPELAND NG, GILBERT DJ, JENKINS N, BARTON D, FRANCKE U, KOBAYASHI Y AND KIM K. (1991). A melanocyte-specific gene, pMel17, maps near the silver coat colour locus on mouse chromosome 10 and is in a syntenic region on human chromosome 12. *Proc. Natl Acad. Sci. USA*, **88**, 9228–9232.

MATTES MJ, THOMSON TM, OLD LJ AND LLOYD KO. (1983). A pigmentation-associated, differentiation antigen of human melanoma defined by a precipitating antibody in human serum. *Int. J. Cancer*, **32**, 717–721.

RUITER DJ. (1990). Melanomas and other skin neoplasms. Pathology and case reports. *Curr. Opin. Oncol.*, **2**, 377–387.

SHILYANSKI J, NISHIMURA MI, YANELLI JR, KAWAKAMI Y, JACKNIN LS, CHARMLY P AND ROSENBERG SA. (1994). T-cell receptor usage by melanoma-specific clonal and highly oligoclonal tumour-infiltrating lymphocytes. *Proc. Natl Acad. Sci. USA*, **91**, 2829–2833.

VAN DER BRUGGEN P, TRAVERSARI C, CHOMEZ P, LURQUIN C, DE PLAEN E, VAN DEN EYNDE B, KNUTH A AND BOON T. (1991). A gene encoding an antigen recognised by cytolytic T lymphocytes on a human melanoma. *Science*, **254**, 1643–1647.

VENNEGOOR C, HAGEMAN P, VAN NOUHUIJS H, RUITER DJ, CALAFAT J, RINGENS PJ AND RÜMKE P. (1988). A monoclonal antibody specific for cells of the melanocytic lineage. *Am. J. Pathol.*, **130**, 179–192.

VIJAYASARADHI S, BOUCHARD B AND HOUGHTON AN. (1990). The melanoma antigen gp75 is the human homologue of the mouse b (brown) locus gene product. *J. Exp. Med.*, **171**, 1375–1380.

VOGEL AM AND ESCLAMADO RM. (1988). Identification of a secreted Mr 95.000 glycoprotein in human melanocytes and melanomas by a melanocyte specific antibody. *Cancer Res.*, **48**, 1286–1294.

WANG RF, ROBBINS PF, KAWAKAMI Y, KANG XQ AND ROSENBERG SA. (1995). Identification of a gene encoding a melanoma tumour antigen recognised by HLA-A31-restricted tumour infiltrating lymphocytes. *J. Exp. Med.*, **181**, 799–804.